# A formula for predicting emphysema extent in combined idiopathic pulmonary fibrosis and emphysema

**DOI:** 10.1186/s12931-023-02589-x

**Published:** 2024-01-18

**Authors:** Athol U. Wells, Joseph Jacob, Nicola Sverzellati, Gary Cross, Joseph Barnett, Angelo De Lauretis, Katerina Antoniou, Derek Weycker, Mark Atwood, Klaus-Uwe Kirchgaessler, Vincent Cottin

**Affiliations:** 1 Royal Brompton Hospital, Sydney Street, London, SW3 6NP UK; 2 Department of Respiratory Medicine, University College London, London, UK; 3 Satsuma Lab, Centre for Medical Image Computing, University College London, London, UK; 4 Scienze Radiologiche, Department of Medicine and Surgery, University Hospital Parma, Parma, Italy; 5 Royal Free Hospital, London, UK; 6 Department of Respiratory Medicine, University of Insubria, Ospedale di Circolo, Varese, Italy; 7 Interstitial Lung Disease Unit, Department of Thoracic Medicine, School of Medicine, University of Crete, Heraklion, Greece; 8 Policy Analysis Inc. (PAI), Brookline, MA USA; 9 F. Hoffmann-La Roche, Ltd., Basel, Switzerland; 10 National Reference Center for Rare Pulmonary Diseases (OrphaLung), Louis Pradel Hospital, Hospices Civils de Lyon, ERN-LUNG, Lyon, France; 11 Université Claude Bernard Lyon 1, Lyon, France

**Keywords:** Clinical trial cohort, Interstitial lung disease, Pulmonary function test, Radiology, Real-world cohort

## Abstract

**Background:**

No single pulmonary function test captures the functional effect of emphysema in idiopathic pulmonary fibrosis (IPF). Without experienced radiologists, other methods are needed to determine emphysema extent. Here, we report the development and validation of a formula to predict emphysema extent in patients with IPF and emphysema.

**Methods:**

The development cohort included 76 patients with combined IPF and emphysema at the Royal Brompton Hospital, London, United Kingdom. The formula was derived using stepwise regression to generate the weighted combination of pulmonary function data that fitted best with emphysema extent on high-resolution computed tomography. Test cohorts included patients from two clinical trials (n = 455 [n = 174 with emphysema]; NCT00047645, NCT00075998) and a real-world cohort from the Royal Brompton Hospital (n = 191 [n = 110 with emphysema]). The formula is only applicable for patients with IPF and concomitant emphysema and accordingly was not used to detect the presence or absence of emphysema.

**Results:**

The formula was: predicted emphysema extent = 12.67 + (0.92 x percent predicted forced vital capacity) – (0.65 x percent predicted forced expiratory volume in 1 second) – (0.52 x percent predicted carbon monoxide diffusing capacity). A significant relationship between the formula and observed emphysema extent was found in both cohorts (R^2^ = 0.25, *P* < 0.0001; R^2^ = 0.47, *P* < 0.0001, respectively). In both, the formula better predicted observed emphysema extent versus individual pulmonary function tests. A 15% emphysema extent threshold, calculated using the formula, identified a significant difference in absolute changes from baseline in forced vital capacity at Week 48 in patients with baseline-predicted emphysema extent < 15% versus ≥ 15% (*P* = 0.0105).

**Conclusion:**

The formula, designed for use in patients with IPF and emphysema, demonstrated enhanced ability to predict emphysema extent versus individual pulmonary function tests.

**Trial registration:**

NCT00047645; NCT00075998.

**Supplementary Information:**

The online version contains supplementary material available at 10.1186/s12931-023-02589-x.

## Background

Idiopathic pulmonary fibrosis (IPF) is a progressive and debilitating lung disease [1, 2]. Emphysema, where the alveoli are damaged and grossly enlarged, is present in approximately 30–40% of patients with IPF [3, 4]. Patients with IPF and emphysema (combined pulmonary fibrosis and emphysema [CPFE]) exhibit characteristics of both fibrosis and emphysema [5, 6].

Identification of emphysema in patients with IPF is important because, compared with IPF alone, patients with IPF and emphysema may have greater lung volumes, yet reduced diffusion capacity [7–16]. An emphysema extent of ≥ 15% has been associated with reduced longitudinal decline in forced vital capacity (FVC) [4]. This has important implications for monitoring patients with IPF, where FVC is a routine variable [17], and for clinical trial design, where FVC is widely used to identify disease progression [18, 19]. Emphysema extent is currently identified on chest high-resolution computed tomography (HRCT) [4]. However, this has limitations, including the requirement for detailed scoring of emphysema extent by a radiologist and interobserver variation [20].

Automated computed tomography (CT) evaluation of interstitial lung disease (ILD), studied in the last decade and applied to trial cohorts, has not been integrated into routine practice, even in expert centres, and is highly unlikely to be available in routine practice in the near future. More importantly, it cannot be applied to emphysema quantification in CPFE. In a recent expert group task force statement, it was stressed that automated CT evaluation does not distinguish between low-density areas due to emphysema and areas of similar low density due to honeycomb cysts or traction bronchiectasis [6]. Thus, automated CT scoring, unlike routine pulmonary function tests (PFTs), is currently an ‘academic tool’ that cannot quantify emphysema in CPFE in real-world practice.

No individual PFT can capture the total functional effect of emphysema in patients with IPF [4]; therefore, other methods are required. We hypothesise that a formula to predict emphysema extent in patients with IPF and emphysema based on composite indices of PFTs may have good accuracy versus HRCT and better accuracy versus individual PFTs. Here we describe the development of such a formula, based on composite indices of percent predicted FVC, percent predicted forced expiratory volume in 1 s (FEV_1_) and percent predicted carbon monoxide diffusing capacity (DLco), and its testing in real-world and clinical trial cohorts of patients with IPF and emphysema. This formula was then used to investigate the relationship between predicted emphysema extent and longitudinal changes in FVC to see if the formula would be consistent with previous findings that a greater emphysema extent on HRCT was associated with reduced FVC decline [4]. Some of the results in this manuscript have been reported previously in abstract form [21].

## Methods

### Development of a formula to predict emphysema extent

#### Development cohort

The development cohort originated from a historical cohort of consecutive patients who presented at the Royal Brompton Hospital (London, United Kingdom) and were diagnosed with IPF between December 1990 and December 1996 [22].

The methodology used to derive the formula was designed to capture patients to whom the formula might be applied in clinical practice or in clinical trial enrolment. In these settings, the formula is only relevant to patients with emphysema observed on HRCT. Therefore, patients without trace of emphysema at visual inspection were excluded, leaving only the patients who exhibited concomitant emphysema of any extent.

#### Derivation of a formula for patients with IPF and emphysema

In the development cohort, presence and extent of emphysema were determined using HRCT and previously published methodology [23]. For each patient, five HRCT sections (origin of the great vessels, mid-arch of the aorta, carina, pulmonary venous confluence, and 1 cm above the right dome of the diaphragm) were scored by two radiologists. Emphysema extent in each section was estimated to the nearest 5% and the score was averaged across the five sections. [4].

PFT data were also collected for each patient, including percent predicted FVC, percent predicted FEV_1_, percent predicted DLco corrected for haemoglobin, total lung capacity, residual volume, carbon monoxide transfer coefficient, and partial pressure of oxygen. Stepwise regression using these PFT variables was performed to generate the weighted combination of the PFT data (co-variables) that fitted best with the emphysema extent on HRCT (dependent variable), to derive a formula that would predict emphysema extent independently of fibrosis extent through calculation of a ‘CPFE Index’; variables that were definitively non-significant in multivariable models were discarded. Similar methodology was previously used to develop the composite physiologic index (CPI), to assess fibrosis extent in ILD independently of emphysema extent [22].

### Testing of the formula to predict emphysema extent

#### Clinical trial test cohort

The formula was tested using data from patients enrolled in two randomised, double-blind, placebo-controlled trials of interferon-γ-1b in patients with IPF: GIPF-001 (NCT00047645) [24] and GIPF-007 (NCT00075998) [25]. Neither trial showed a treatment effect on progression-free survival, pulmonary function, quality of life or mortality, allowing patients from the treatment and placebo groups to be combined in this analysis.

For the analyses described here, data were limited to those from patients considered in a previous *post-hoc* evaluation of emphysema extent and serial lung function [4]. Patients were required to have HRCT images available from baseline, and data from Week 48 of the relevant trial. It was not possible to review HRCT images for all patients who were eligible for inclusion in this analysis; the sample was limited to all patients with FEV_1_/FVC ratio < 0.8 or > 0.9 in GIPF-001 or < 0.7 or > 0.9 in GIPF-007, and randomly selected patients with FEV_1_/FVC ratios 0.7–0.8 (GIPF-007 only) and 0.8–0.9 (GIPF-001 and GIPF-007). Only eight patients enrolled in GIPF-001 had an FEV_1_/FVC ratio < 0.7, so different inclusion criteria were used to select patients compared with GIPF-007.

#### Real-world test cohort

The formula was also tested in a more current population of consecutive patients who presented at the Royal Brompton Hospital and received a multidisciplinary diagnosis of IPF between January 2011 and June 2014. Patients were required to have volumetric HRCTs and concurrent PFTs.

#### Testing of the formula in test cohorts with IPF and emphysema

In the clinical trial test cohort, all patients had emphysema extent on HRCT scored using the same methodology as that employed for the development cohort [4, 23]. Where present, emphysema was additionally quantified as occurring separately from (isolated) and within (admixed) areas of fibrosis.

In the real-world test cohort, emphysema and fibrosis extent were scored on a lobar basis, with the lingula considered a separate lobe. The total amount of emphysema and fibrosis was estimated as a percentage of the lobar volume. The sum of the percentages for each of the six lobes was then averaged to derive a total lung percentage for emphysema and fibrosis.

In the clinical trial test cohort, the relationship between the predicted emphysema extent using the formula and the observed emphysema extent on HRCT was investigated using linear regression analyses (with calculation of the coefficient of determination, R^2^ and *P*-value) and Pearson’s correlation (with calculation of R and *P*-value). *P*-values were based on Student’s t distribution with n-2 degrees of freedom. This analysis was repeated in the real-world test cohort.

These analyses were performed using data from patients with IPF and concomitant emphysema, and also patients with IPF, with and without emphysema.

Relationships between individual PFTs and the observed emphysema extent on HRCT were investigated using Pearson product-moment correlations. This analysis was performed for both the clinical trial test cohort and the real-world test cohort, and included only patients with IPF and concomitant emphysema.

#### Relationship between predicted emphysema extent and change in FVC

Linear mixed-effect model analysis with random intercept and slope at the patient level was conducted to explore the relationships between the predicted emphysema extent and changes in FVC in all patients with IPF, with and without emphysema. In the clinical trial test cohort, FVC change was considered from baseline to Week 48, whereas in the real-world cohort, all available FVC measurements were used to consider change from baseline. Various models were tested, each with three versions building on the previous (A = basic [CPFE Index + months]; B = basic + demographics [ILD extent, sex, age, smoking status]; C = basic + demographics + interaction factor [CPFE Index x ILD extent]).

Baseline FVC (L) and relative (%) and absolute (L) changes from baseline in FVC over 48 weeks were analysed in the clinical trial test cohort (including patients with and without emphysema) by decile of predicted emphysema extent (1st − 10th deciles of CPFE Index).

Based on previous findings that an emphysema extent ≥ 15% scored on HRCT may be associated with reduced longitudinal decline in FVC [4], relative and absolute changes from baseline in FVC over 48 weeks were analysed for patients in the clinical trial test cohort (including patients with and without emphysema) by baseline-predicted emphysema extent < 15% and ≥ 15%, calculated using the CPFE Index.

## Results

### Patient populations

Of 212 patients from the historical cohort of patients with IPF, 76 (35.8%) patients who exhibited concomitant emphysema were included in the development cohort. The test populations included 455 patients in the clinical trial test cohort, of whom 174 patients had concomitant emphysema (38.2%), and 191 patients in the real-world test cohort, of whom 110 patients had concomitant emphysema (57.6%).

In the clinical trial test cohort, the single determinant standard deviation (SDSD) for extent of emphysema (as agreed by both observers) was 3.55% and mean emphysema extent ranged from 0 to 32.5%. In the real-world test cohort, the SDSD for extent of emphysema (as agreed by both observers) was 2.89% and mean emphysema extent ranged from 0 to 51%.

Demographics and clinical characteristics for the patient populations with IPF and emphysema are shown in Table 1. Pulmonary function was generally similar across the three patient populations. The extent of fibrosis was higher in the development cohort and the clinical trial cohort versus the real-world cohort (mean [standard deviation (SD)] 49.3% [19.7], 41.2% [13.9] and 27.9% [10.9], respectively). Emphysema extent was similar in the clinical trial and real-world test cohorts (mean [SD] 13.1% [13.7] and 11.6% [12.2], respectively) but higher in the development cohort (mean [SD] 18.3% [17.3]). Demographics and clinical characteristics for all patients with IPF, with and without emphysema, are presented in the Online Supplement (Table S1).

**Table 1:** Demographics and clinical characteristics for patient populations with IPF and emphysema

Characteristic*	Development cohort (N = 76)	Clinical trial test cohort (N = 174)	Real-world test cohort (N = 110)
Male sex, *n* (%)	66 (86.8)	146 (83.9)	91 (82.7)
Smoking status, *n* (%)			
Current	14 (18.4)^‡^	22 (12.6)	1 (0.9)^§^
Former	58 (76.3)^‡^	98 (56.3)	88 (80.0)^§^
Age, years	62.3 (10.7)	64.6 (8.6)	69.7 (8.2)
FVC, % predicted	81.7 (21.1)	75.9 (13.4)	74.9 (18.2)
FEV_1_, % predicted	78.2 (18.6)	76.0 (12.8)	76.4 (15.9)
Corrected DLco^||^, % predicted	36.9 (14.4)	41.1 (10.0)	34.9 (13.8)
CPI	50.4 (14.2)	49.9 (8.8)	54.6 (12.3)
Fibrosis extent, %	49.3 (19.7)	41.2 (13.9)	27.9 (10.9)
Isolated emphysema extent, %	7.3 (6.1)	6.8 (8.6)	6.1 (8.6)
Admixed emphysema extent, %	11.0 (15.6)	6.3 (7.0)	5.5 (5.3)
Total emphysema extent, %	18.3 (17.3)	13.1 (13.7)	11.6 (12.2)
CPFE Index	17.9 (12.8)	12.1 (6.9)	13.8 (10.5)

*CPFE* combined pulmonary fibrosis and emphysema; *CPI* composite physiologic index; *DLco* carbon monoxide diffusing capacity; *FEV*_*1*_ forced expiratory volume in 1 s; *FVC* forced vital capacity; *IPF* idiopathic pulmonary fibrosis; *SD* standard deviation

* Data are mean (SD) unless otherwise specified

^‡^ One patient in the development cohort had insufficient data to conclude smoking status

^§^ Two patients in the real-world test cohort had insufficient data to conclude smoking status

^||^ Corrected for haemoglobin

### Derived formula for patients with IPF and emphysema using the development cohort

The derived formula, calculated using the development cohort of patients with IPF and emphysema (n = 76), was: predicted emphysema extent (CPFE Index) = 12.67 + (0.92 x percent predicted FVC) – (0.65 x percent predicted FEV_1_) – (0.52 x percent predicted corrected DLco). A negative CPFE Index value indicates the expected absence of emphysema and should be replaced by zero.

### Relationship between CPFE Index and observed emphysema extent in the test cohorts

**Fig. 1:**
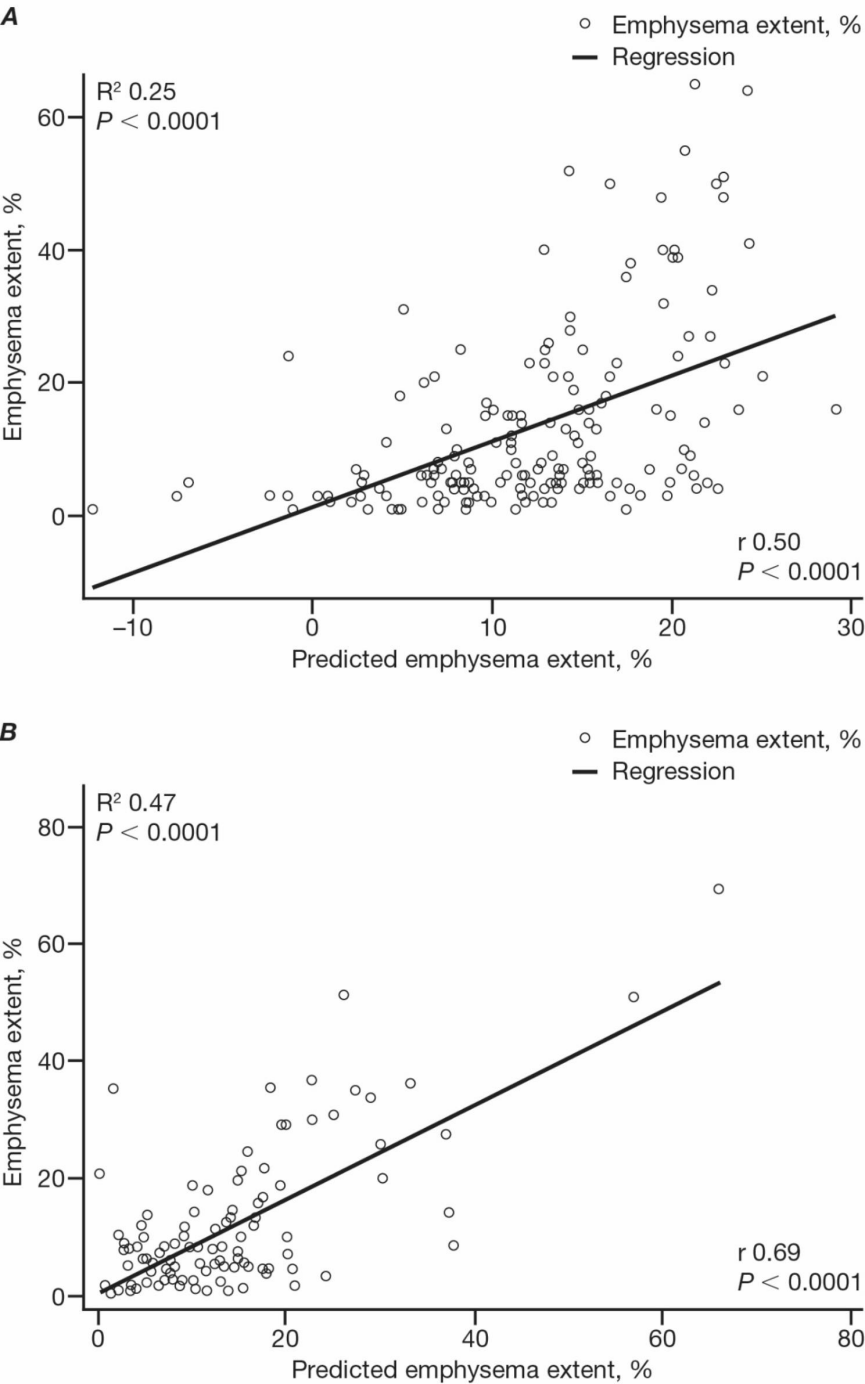
Correlation between CPFE Index and emphysema extent Correlation between predicted emphysema extent (CPFE Index) and observed emphysema extent (on HRCT) for patients with IPF and emphysema in (**A**) the clinical trial test cohort and (**B**) the real-world test cohort. *CPFE* combined pulmonary fibrosis and emphysema; *HRCT* high-resolution computed tomography; *IPF* idiopathic pulmonary fibrosis *CPFE*, combined pulmonary fibrosis and emphysema; *HRCT*, high-resolution computed tomography; *IPF*, idiopathic pulmonary fibrosis

In the clinical trial test cohort of patients with IPF and emphysema (n = 174), a significant relationship was found between the CPFE Index and observed emphysema extent on HRCT (R^2^ = 0.25; *P* < 0.0001) (Fig. 1A). In the real-world test cohort of patients with IPF and emphysema (n = 110), a stronger significant relationship was found between the CPFE Index and observed emphysema extent on HRCT (R^2^ = 0.47; *P* < 0.0001) (Fig. 1B) than in the clinical trial test cohort. The relationship between the CPFE Index and observed emphysema extent was also investigated in all patients with IPF, with and without emphysema, in the clinical trial cohort (n = 455; Fig. S1A and Table S2) and real-world test cohort (n = 191; Fig. S1B).

In both test cohorts of patients with IPF and emphysema (clinical trial test cohort: n = 174; real-world test cohort: n = 110), the CPFE Index better predicted observed emphysema extent on HRCT compared with the individual PFT data (Table 2).

**Table 2:** Correlation between PFTs and observed emphysema extent (on HRCT) for patients with IPF and emphysema

	Development cohort (N = 76)		Clinical trial test cohort (N = 174)		Real-world test cohort (N = 110)	
	R^2^	*P*-value	R^2^	*P*-value	R^2^	*P*-value
CPFE Index	0.55	< 0.0001	0.25	< 0.0001	0.47	< 0.0001
FVC, % predicted	0.14	< 0.001	0.08	0.0001	0.09	0.0012
FEV_1_, % predicted	0.01	0.53	0.01	0.33	0.001	0.71
Corrected DLco*, % predicted	0.03	0.13	0.01	0.17	0.12	0.0002
FVC/corrected DLco* ratio	0.24	< 0.0001	0.09	< 0.0001	0.45	< 0.0001

*CPFE* combined pulmonary fibrosis and emphysema; *DLco* carbon monoxide diffusing capacity; *FEV*_*1*_ forced expiratory volume in 1 s; *FVC* forced vital capacity;

*HRCT* high-resolution computed tomography; *IPF* idiopathic pulmonary fibrosis; *PFT* pulmonary function test; *R*^*2*^ explained variation/total variation

For R^2^ of 0 to 1, the closer to 1, the better the regression prediction fits the data

* Corrected for haemoglobin

### Relationship between predicted emphysema extent and change in FVC

Linear mixed-effect model analysis explored the relationships between predicted emphysema extent and relative and absolute changes from baseline in FVC in patients with IPF, with and without emphysema (Table 3). In the clinical trial test cohort (n = 455), the relationship between the CPFE Index and relative FVC change over 48 weeks was significant in Model A (basic), but not in Models B (basic + demographics) or C (basic + demographics + interaction factor). For absolute FVC change over 48 weeks, there was no significant relationship between CPFE Index and FVC change across any of the models in the clinical trial test cohort. In the real-world test cohort using all available data (mean follow-up of 18.8 months), Model C (basic + demographics + interaction factor) showed the strongest association with relative and absolute FVC change (coefficient [relative FVC change] = − 0.02; *P* = 0.0259 and coefficient [absolute FVC change] = − 0.03; *P* = 0.0649). When the linear mixed-effect model analysis was performed using the CPFE Index as a dichotomous variable (< 15% versus ≥ 15%) instead of a continuous variable, the relationship between the CPFE Index and relative or absolute FVC change over 48 weeks in the clinical trial test cohort was significant in all models (Table 4).

**Table 3:** Linear mixed-effect models for change in FVC over time

	Clinical trial cohort (N = 455)				Real-world cohort* (N = 124)			
	Relative FVC change (%)		Absolute FVC change (L)		Relative FVC change (%)		Absolute FVC change (L)	
	Coefficient	*P*-value	Coefficient	*P*-value	Coefficient	*P*-value	Coefficient	*P*-value
**Model A**								
CPFE formula	0.0646	< 0.0001	0.0012	0.2445	0.0025	0.3708	0.0048	0.3686
Months^‡^	–0.1143	< 0.0001	–0.0030	< 0.0001	–0.0042	< 0.0001	–0.0108	0.0000
**Model B**								
CPFE formula	0.0362	0.2564	0.0009	0.2934	0.0007	0.7964	0.0020	0.7061
Months^‡^	–0.1087	< 0.0001	–0.0026	< 0.0001	–0.0042	< 0.0001	–0.0108	0.0000
ILD extent	–0.0533	0.0012	–0.0013	0.0016	0.0045	0.0221	0.0064	0.0781
Male	–0.4328	0.3787	–0.0203	0.1111	–0.1087	0.0583	–0.1681	0.1121
Age	0.0312	0.2410	0.0009	0.2115	0.0058	0.0112	0.0145	0.0005
Current/former smoker	0.3737	0.4488	0.0093	0.4627	0.0935	0.0340	0.1134	0.1652
**Model C**								
CPFE formula	0.0841	0.3546	0.0025	0.2832	–0.0214	0.0259	–0.0333	0.0649
Months^‡^	–0.1085	< 0.0001	–0.0026	< 0.0001	–0.0042	< 0.0001	–0.0108	0.0000
ILD extent	–0.0425	0.0920	–0.0010	0.1370	0.0004	0.8762	–0.0001	0.9858
Male	–0.4327	0.3791	–0.0203	0.1114	–0.1099	0.0501	–0.1696	0.1033
Age	0.0310	0.2440	0.0009	0.2150	0.0062	0.0055	0.0152	0.0002
Current/former smoker	0.3861	0.4340	0.0098	0.4423	0.0907	0.0354	0.1084	0.1778
CPFE formula x ILD extent	–0.0012	0.5732	0.0000	0.4514	0.0009	0.0161	0.0015	0.0412

Linear mixed-effects models for relative (%) and absolute (L) change from baseline in FVC over 48 weeks in the clinical trial cohort and all available data* for the real-world cohort for patients with IPF, with and without emphysema

*CPFE* combined pulmonary fibrosis and emphysema; *FVC* forced vital capacity; *ILD* interstitial lung disease; *IPF* idiopathic pulmonary fibrosis

* Mean follow-up time of 18.8 months

^‡^ Time elapsed from baseline lung-function tests

**Table 4:** Linear mixed-effect models for change in FVC over 48 weeks using CPFE dichotomous at 15%

	Clinical trial cohort (N = 455)			
	Relative change in FVC (%)		Absolute change in FVC (L)	
	Coefficient	*P*-value	Coefficient	*P*-value
**Model A**				
CPFE formula ≥ 15%	2.43	0.0009	0.06	0.0024
Months	–0.11	< 0.0001	–0.003	< 0.0001
**Model B**				
CPFE formula ≥ 15%	1.61	0.0048	0.04	0.0027
Months	–10.90	< 0.0001	–0.003	< 0.0001
ILD extent	–0.05	0.0033	–0.001	0.0053
Male	–0.35	0.48	–0.02	0.16
Age	0.03	0.22	0.001	0.19
Current/former smoker	0.29	0.56	0.01	0.58
**Model C**				
CPFE formula ≥ 15%	1.98	0.0045	0.06	0.0016
Months	–0.11	< 0.0001	–0.003	< 0.0001
ILD extent	–0.04	0.0138	–0.001	0.0262
Male	–0.34	0.49	–0.02	0.16
Age	0.03	0.24	0.001	0.21
Current/former smoker	0.27	0.59	0.01	0.62
CPFE formula x ILD extent	–0.001	0.35	0.0000	0.21

Linear mixed-effects models for relative (%) and absolute (L) change from baseline in FVC over 48 weeks for patients with IPF, with and without emphysema, in the clinical trial cohort (N = 455) using CPFE dichotomous at 15%

*CPFE* combined pulmonary fibrosis and emphysema; *FVC* forced vital capacity; *ILD* interstitial lung disease; *IPF* idiopathic pulmonary fibrosis

In an analysis of relative (%) and absolute (L) changes in FVC from baseline at Week 48 by decile of predicted emphysema extent in the clinical trial test cohort (including all patients with and without emphysema; n = 455), a difference in FVC change was observed between patients in deciles 1–9 (corresponding to predicted emphysema extent at baseline of ≤ 18.7%) versus patients in decile 10 (corresponding to predicted emphysema extent at baseline of 19.2–29.7%) (Table 5). In deciles 1–9, a mean reduction from baseline in both relative and absolute FVC was observed at Week 48 (mean FVC change ranged from −2.6% to −8.8% for relative change and from −0.07 L to −0.23 L for absolute change). In decile 10, a smaller mean decrease in both relative and absolute FVC was seen (–0.8% and −0.02 L for relative and absolute FVC change, respectively).

**Table 5:** Changes in FVC at Week 48 by decile of predicted emphysema extent (clinical trial cohort)

Decile of predicted emphysema extent	*N*	Median predicted emphysema extent, %	Mean (SD) FVC, L	Change in mean (SD) FVC from baseline	
				Relative, %	Absolute, L
Total	455	8.4	2.80 (0.78)	–5.4 (12.1)	–0.14 (0.34)
1st	45	–4.5	2.48 (0.68)	–5.7 (14.2)	–0.12 (0.32)
2nd	46	0.3	2.57 (0.69)	–5.5 (12.3)	–0.15 (0.32)
3rd	45	3.1	2.72 (0.81)	–8.8 (13.1)	–0.22 (0.34)
4th	46	5.3	2.65 (0.69)	–4.9 (11.5)	–0.15 (0.31)
5th	45	7.6	2.67 (0.50)	–7.0 (9.5)	–0.18 (0.23)
6th	46	9.2	2.76 (0.76)	–4.6 (12.8)	–0.12 (0.32)
7th	46	11.3	2.74 (0.71)	–6.2 (12.8)	–0.15 (0.68)
8th	45	13.4	3.03 (0.87)	–7.6 (9.6)	–0.23 (0.30)
9th	46	15.9	3.12 (0.90)	–2.6 (10.2)	–0.07 (0.34)
10th	45	21.0	3.21 (0.80)	–0.8 (12.7)	–0.02 (0.46)

Relative (%) and absolute (L) changes in FVC from baseline at Week 48 for patients with IPF, with and without emphysema, in the clinical trial cohort (N = 455) by decile of predicted emphysema extent

*FVC* forced vital capacity; *IPF* idiopathic pulmonary fibrosis; *SD* standard deviation

In an analysis of relative and absolute changes in FVC from baseline at Week 48 by emphysema extent < 15% versus ≥ 15% in the clinical trial test cohort (including all patients with and without emphysema; n = 455), the mean (SD) relative changes from baseline in FVC at Week 48 for patients with predicted emphysema extent < 15% versus ≥ 15% were −6.3% (12.0) versus −1.4% (11.8) (*P* = 0.0008; Table 6). For absolute changes from baseline in FVC at Week 48, the mean (SD) changes for patients with predicted emphysema extent < 15% versus ≥ 15% were −0.16 L (0.31) versus −0.04 L (0.41) (*P* = 0.0105; Table 6).

**Table 6:** Baseline FVC and changes in FVC at Week 48 by emphysema extent < 15%/≥ 15%

Predicted emphysema extent	Clinical trial cohort (N = 455)				
	*N*	Median predicted emphysema extent,%	Mean (SD) FVC, L	Change in mean (SD) FVC frombaseline	
				Relative, %	Absolute, L
Baseline	455	8.4	2.80 (0.78)	–	–
< 15%	372	6.7	2.71 (0.74)	–	–
≥ 15%	83	19.5	3.20 (0.82)	–	–
Week 48	443	9.0	2.69 (0.83)	–5.4 (12.1)	–0.14 (0.34)
< 15%	349	7.1	2.56 (0.75)	–6.3 (12.0)	–0.16 (0.31)
≥ 15%	94	19.9	3.14 (0.95)	–1.4 (11.8)	–0.04 (0.41)
				*P* = 0.0008*	*P* = 0.0105^‡^

Baseline FVC (L) and changes from baseline in relative (%) and absolute (L) FVC at Week 48 by emphysema extent < 15% and ≥ 15% for patients with IPF, with and without emphysema, in the clinical trial cohort (N = 455)

*FVC* forced vital capacity; *IPF* idiopathic pulmonary fibrosis; *SD* standard deviation

* Comparison of change in FVC from baseline to Week 48 between the < 15% and ≥ 15% baseline-predicted emphysema extent groups using a t-test with equal variance

^‡^ Comparison of change in FVC from baseline to Week 48 between the < 15% and ≥ 15% baseline-predicted emphysema extent groups using a t-test with unequal variance

## Discussion

Here, we describe a composite CPFE Index based on PFTs, validated in clinical trial and real-world patient cohorts, that can quantify emphysema extent in patients with IPF and emphysema, and that correlates much more strongly than individual PFTs with emphysema extent on CT. We confirmed previous findings that visually scored emphysema extent ≥ 15% is associated with reduced FVC decline over 48 weeks versus emphysema extent < 15% [4], both in the same clinical trial population as the previous analysis and in a separate real-world cohort. Our findings also confirm that emphysema extent ≥ 15% has a functional effect on longitudinal FVC, and suggest that the CPFE Index is as effective as visual scoring of emphysema extent in IPF on HRCT at predicting the impact of emphysema on FVC decline.

By using the CPFE Index to predict emphysema extent in patients with IPF and emphysema, and application of the ≥ 15% emphysema extent threshold, clinicians can immediately identify patients in whom emphysema will likely have a functional impact and longitudinal FVC may not adequately capture disease progression. The CPFE Index requires only routine PFTs, is easy to generate, reduces the need for detailed scoring of emphysema extent on chest HRCTs by experienced thoracic radiologists, reduces the risk of interobserver variability, and is particularly beneficial when radiological input is unavailable. Although not a perfect fit, the R^2^ values (0.25 and 0.47 for the clinical trial and real-world cohort, respectively) provide the best fit and allow lung function tests to be used to quantify emphysema; common to many studies using PFT, confounding effects of the normal PFT range and variation of PFT results between centres may have influenced the fit. Looking to the future for new strategies, recent studies have suggested that artificial intelligence may be a valuable tool in diagnosing ILDs, though this requires further validation [26, 27]. In addition, as discussed earlier, automated methodologies are poorly suited to emphysema quantification in CPFE, as honeycombing or traction bronchiectasis typically co-exist with emphysema and can result in major emphysema misclassification. Until this problem is overcome, a computational threshold of emphysema, above which emphysema has a functional effect on longitudinal FVC, will not be achievable.

In clinical trials, change from baseline in FVC remains the most common primary endpoint in patients with IPF (e.g., NCT03955146, NCT04419558, NCT04965298, NCT04552899, NCT04708782). However, our results demonstrate that this endpoint is not suitable in patients with ≥ 15% emphysema extent, who will typically have increased lung volume and reduced longitudinal FVC decline. The CPFE Index may present an easy way to identify patients with ≥ 15% emphysema extent and allow for these patients to be excluded from IPF clinical trials; or the ≥ 15% threshold could be used to stratify randomisation to ensure balanced distribution between treatment arms. Alternatively, the formula may help enrich study populations with patients at risk of FVC decline. Whilst the CPFE Index was more strongly correlated with emphysema extent on HRCT compared with individual PFT variables in all cohorts, the strength of the correlation was reduced in the clinical trial cohort, potentially due to greater inter-site variability in PFT measurement, as well as differences between real-world and clinical trial populations.

It should be noted that the CPFE Index is only applicable for patients with IPF and concomitant emphysema and should not be applied in patients with non-IPF ILD. It should not be applied to detect the presence or absence of emphysema; rather, the presence of emphysema would need to be identified on HRCT, and the formula would be used to predict the functional impact of the emphysema. Although there may be a correlation between emphysema extent on HRCT and functional impact of emphysema in PFTs, the formula is a composite with different weightings to individual PFTs, so a 15% extent on HRCT does not mean a 15% impairment in PFTs due to emphysema. It should also be noted that it is possible for the formula to result in negative values due to the statistical assumption when deriving the formula that all patients started with 100% predicted lung function, whereas, in reality, this is not the case. If the formula results in a negative value in a patient with emphysema detected on HRCT, it should be considered that emphysema has no determinable functional impact.

A similar methodology to that used to develop the CPFE Index was used to develop the CPI, a formula designed to predict fibrosis extent independently of emphysema extent in patients with IPF [22]. The CPI correlated more strongly with fibrosis extent compared with individual PFTs and was also linked to mortality.

The current analyses have several limitations. Firstly, it should be acknowledged that the ‘measurement error’ from scoring emphysema on HRCT is replaced by ‘measurement error’ from using percent predicted values of lung function, since premorbid ‘normal’ PFT values in individual patients are almost always unknown. Secondly, patient cohorts from academic institutions, such as the Royal Brompton Hospital, are not fully representative of a real-world cohort. Additionally, the long period of time that elapsed between the selection of the patient cohorts included in this analysis could have affected the results due to differences in imaging techniques and diagnostic criteria. For example, a different method was used to score emphysema in the development cohort and the clinical trial test cohort, compared with the real-world test cohort. In the development cohort and the clinical trial test cohort, non-volumetric interspaced CT imaging was standard, and individual CT slices were scored for the presence of emphysema, whilst in the real-world test cohort, lobar scores of emphysema extent were possible. Diagnostic criteria for IPF have also changed over time [2, 17], and consistency of IPF diagnoses across the cohorts could be questioned, particularly for the development cohort (although the demographic data of the development cohort are consistent with IPF). Ultimately, it is important to consider the potential impact of these differences when interpreting the results. The development and validation of the formula across populations with such differences can be considered a strength, demonstrating the validity and wide applicability of the formula. Further, the relationship between predicted emphysema extent and FVC change was evaluated over 48 weeks in the clinical trial cohort, whereas only 51 patients in the real-world test cohort had at least 48 weeks’ follow-up. However, the mixed-effects model allows for analysis of a range of follow-up times (as would be the case in normal clinical practice).

Pulmonary vasculopathy can contribute to the decline of DLco in patients with IPF, and may be a source of variability in the CPFE Index, which could lead to inaccurate predictions of emphysema extent. This may be particularly relevant in patients with IPF and pulmonary hypertension (PH). Future studies applying the CPFE Index to patients with IPF, split by PH, may be required to identify whether pulmonary vasculopathy impacts the results of the CPFE Index in these patients and if a separate formula may be required for patients with IPF and PH.

## Conclusion

In conclusion, the CPFE Index predicts the extent of emphysema on HRCT in patients with IPF regardless of the extent or severity of fibrosis. The CPFE Index addresses the need to quantify the functional impact of emphysema in IPF in clinical practice and may help identify patients in whom longitudinal measurement of FVC will not adequately capture disease progression. In clinical trials, the CPFE Index could be used to develop eligibility criteria, stratify randomisation, or enrich study populations.

### Electronic supplementary material

Below is the link to the electronic supplementary material.

Supplementary Material 1

## Data Availability

Qualified researchers may request access to individual patient-level data through the clinical study data request platform (https://vivli.org/). Further details on Roche’s criteria for eligible studies are available here (https://vivli.org/members/ourmembers/). For further details on Roche’s Global Policy on the Sharing of Clinical Information and how to request access to related clinical study documents, see here (https://www.roche.com/research_and_development/who_we_are_how_we_work/clinical_trials/our_commitment_to_data_sharing.htm).

## Abbreviations

CPFE: Combined pulmonary fibrosis and emphysema
CPI: Composite physiologic index
CT: Computed tomography
DLco: Carbon monoxide diffusing capacity
FVC: Forced vital capacity
FEV_1_: Forced expiratory volume in 1 s
HRCT: High-resolution computed tomography
ILD: Interstitial lung disease
IPF: Idiopathic pulmonary fibrosis
PFT: Pulmonary function test
PH: Pulmonary hypertension
SD: Standard deviation
SDSD: Single determinant standard deviation
