# Supplementary material for: A formula for predicting emphysema extent in combined idiopathic pulmonary fibrosis and emphysema
Source: Respir Res. 2024 Jan 18;25:33. doi: 10.1186/s12931-023-02589-x (PMC10795205; doi:10.1186/s12931-023-02589-x)
Supplement: Supplementary file 1 — Supplementary Material 1 [file 12931_2023_2589_MOESM1_ESM.pdf]

Online Supplement

for

A Formula for Predicting Emphysema Extent in Combined Idiopathic Pulmonary Fibrosis and Emphysema

Athol U. Wells, Joseph Jacob, Nicola Sverzellati, Gary Cross, Joseph Barnett, Angelo De Lauretis, Katerina Antoniou, Derek Weycker, Mark Atwood, Klaus-Uwe Kirchgaessler, Vincent Cottin

Supplementary Results

Further analyses in all patients with idiopathic pulmonary fibrosis (IPF), with and without emphysema

Patient demographics and clinical characteristics for all patients with IPF, with and without emphysema, for the historical cohort (n = 212; origin of the development cohort), the clinical trial test cohort (n = 455) and the real-world test cohort (n = 191) are shown in Table S1. The prevalence of emphysema was similar in the historical and clinical trial test cohorts (35.8% and 38.2%, respectively). In comparison, the prevalence of emphysema was higher in the real-world test cohort (57.6%). Pulmonary function was generally similar across the three patient populations. There was some variance in the extent of fibrosis, with the highest disease extent observed in the historical cohort (54.6%), followed by the clinical test cohort (45.1%), then the real-world test cohort (28.9%).

In the clinical trial test cohort of patients with IPF, with and without emphysema (n = 455), a significant relationship was observed between the combined pulmonary fibrosis and emphysema (CPFE) Index and observed emphysema extent on high-resolution computed tomography (HRCT) ($R^2 = 0.21$; $P < 0.0001$) (Fig. S1A). This relationship was weaker compared with that found in the clinical trial test cohort that included only patients with emphysema (n = 174). In the real-world test cohort of patients with IPF, with or without emphysema (n = 191), a significant relationship was observed between the CPFE Index and observed emphysema extent on HRCT ($R^2 = 0.46$; $P < 0.0001$) (Fig. S1B).

Table S1. Demographics and clinical characteristics for the cohorts of patients with IPF, with and without emphysema

Characteristic[*]	Historical cohort (origin of development cohort) (N = 212)	Clinical trial test cohort (N = 455)	Real-world test cohort (N = 191)
Male sex, *n* (%)	158 (74.5)	321 (70.5)	154 (80.6)
Smoking status, *n* (%)			
Current	18 (8.7)[‡]	29 (6.4)	1 (0.5)[§]
Former	143 (69.1)[‡]	216 (47.5)	134 (70.2)[§]
Age, years	62.2 (10.6)	65.1 (8.4)	69.6 (8.7)
FVC, % predicted	69.9 (22.1)	70.4 (13.3)	70.7 (18.5)
FEV_1, % predicted	71.2 (19.2)	73.9 (13.2)	73.9 (16.7)
Corrected DLco[||], % predicted	37.5 (15.4)	41.4 (11.0)	35.9 (13.9)
CPI	–	51.9 (9.1)	55.3 (12.3)
Fibrosis extent, %	54.6 (21.2)	45.1 (15.2)	28.9 (11.3)

Prevalence of emphysema, *n* (%)	76 (35.8)	174 (38.2)	110 (57.6)

CPI composite physiologic index; *DLco* carbon monoxide diffusing capacity; FEV_1 forced expiratory volume in 1 second; *FVC* forced vital capacity; *IPF* idiopathic pulmonary fibrosis; *SD* standard deviation.

[*] Data are presented as mean (SD) unless otherwise specified.

[‡] Five patients in the development cohort had insufficient data to conclude smoking status.

[§] Two patients in the real-world test cohort had insufficient data to conclude smoking status.

[||] Corrected for haemoglobin.

Table S2. 2x2 contingency table of predicted versus observed emphysema extent (on HRCT) < 15% versus ≥ 15% in the clinical trial test cohort

N **(%)**	**Predicted emphysema extent**		
**Observed emphysema extent**	**< 15%**	**≥ 15%**	**Total**
< 15%	350 (76.9)	52 (11.4)	402 (88.4)
≥ 15%	22 (4.8)	31 (6.8)	53 (11.7)
Total	372 (81.8)	83 (18.2)	455 (100)

HRCT high-resolution computed tomography.

Fisher's exact test: $P < 0.0001$.

Fig. S1. Correlation between predicted emphysema extent (CPFE Index) and observed emphysema extent (on HRCT) for all patients with IPF, with and without emphysema, in (*A*) the clinical trial test cohort and (*B*) the real-world test cohort

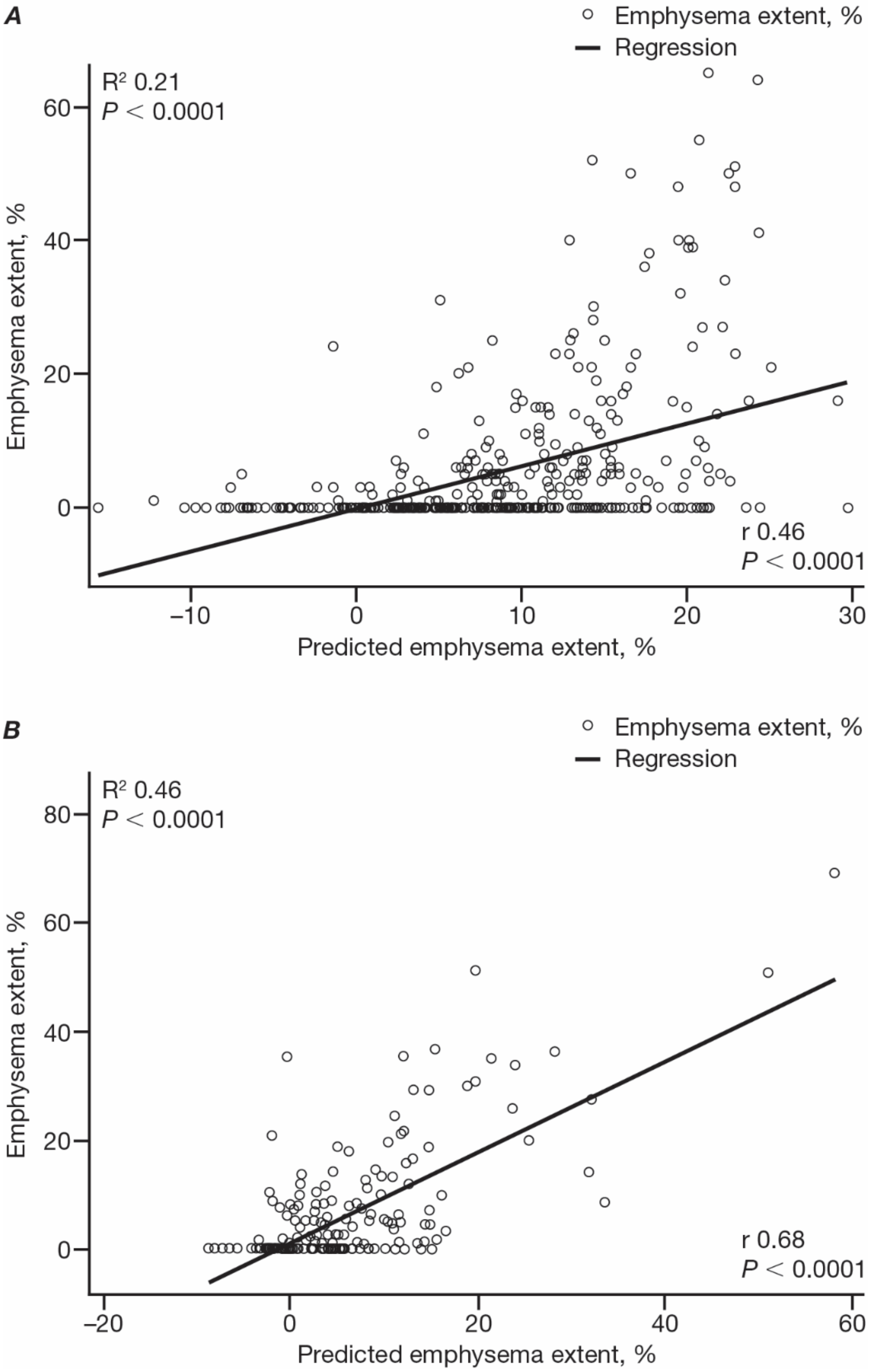

CPFE combined pulmonary fibrosis and emphysema; *HRCT* high-resolution computed tomography; *IPF* idiopathic pulmonary fibrosis.
